# DVDR-SRGAN: Differential Value Dense Residual Super-Resolution Generative Adversarial Network

**DOI:** 10.3390/s23104854

**Published:** 2023-05-18

**Authors:** Hang Qu, Huawei Yi, Yanlan Shi, Jie Lan

**Affiliations:** 1 School of Electronics and Information Engineering, Liaoning University of Technology, Jinzhou 121001, China; 2 College of Science, Liaoning University of Technology, Jinzhou 121001, China

**Keywords:** image reconstruction, super resolution, generative adversarial network, differential value dense residual network, joint loss function

## Abstract

In the field of single-image super-resolution reconstruction, GAN can obtain the image texture more in line with the human eye. However, during the reconstruction process, it is easy to generate artifacts, false textures, and large deviations in details between the reconstructed image and the Ground Truth. In order to further improve the visual quality, we study the feature correlation between adjacent layers and propose a differential value dense residual network to solve this problem. We first use the deconvolution layer to enlarge the features, then extract the features through the convolution layer, and finally make a difference between the features before being magnified and the features after being extracted so that the difference can better reflect the areas that need attention. In the process of extracting the differential value, using the dense residual connection method for each layer can make the magnified features more complete, so the differential value obtained is more accurate. Next, the joint loss function is introduced to fuse high-frequency information and low-frequency information, which improves the visual effect of the reconstructed image to a certain extent. The experimental results on Set5, Set14, BSD100, and Urban datasets show that our proposed DVDR-SRGAN model is improved in terms of PSNR, SSIM, and LPIPS compared with the Bicubic, SRGAN, ESRGAN, Beby-GAN, and SPSR models.

## 1. Introduction

Super-resolution (SR) reconstruction of images is a technique for obtaining high-resolution images from single or multiple low-resolution images. In real life, limited by factors such as digital imaging equipment and hardware storage capacity, the image resolution obtained cannot meet people’s needs, especially in some specific fields such as surveillance, remote sensing, military, medicine, and so on. Using super-resolution image reconstruction technology to restore and reconstruct low-resolution images can efficiently improve image details and image quality.

Image reconstruction methods can be roughly divided into three categories, which are interpolation-based [1], reconstruction-based [2], and learning-based [3]. With the development of the times, the two methods based on interpolation and reconstruction have some shortcomings, such as reconstructing the image blur, insufficient computational power, and serious detail loss. In recent years, the development of deep learning has made the learning-based image reconstruction technology gradually become a research hotspot, and interpolation-based and reconstruction-based technologies are usually used to preprocess images.

In recent years, with the rapid rise of artificial intelligence, image super-resolution reconstruction methods based on the convolutional neural network (CNN) and generative adversarial network (GAN) have been widely used because their reconstruction performance far exceeds that of traditional algorithms. Dong et al. [4] proposed the super-resolution convolutional neural network (SRCNN), which uses three convolutional layers for reconstruction and greatly improves the speed of reconstruction compared to traditional methods. Kim et al. [5] studied SRCNN and constructed a reconstruction model with deep-level networks, which used VGG network architecture to construct a deep convolution network with 20 layers and greatly improved the network convergence speed via a very high learning rate during the training process. Jiang et al. [6] proposed a new differential network in image super-resolution reconstruction to increase the image reconstruction effect and used the multi-path supervised reconstruction blocks to monitor the reconstruction process, making the network more stable. Lim et al. [7] proposed an enhanced depth super-resolution network (EDSR), which removes unnecessary batch normalization (BN) layers in the residual blocks, resulting in a significant performance improvement in the reconstruction. Zhang et al. [8] proposed RCAN, which constructed channel attention to focus on improving the PSNR value. Hu et al. [9] proposed Meta-SR to achieve the effect of up-sampling images to arbitrary sizes. Li et al. [10] proposed a feedback framework for gradually refining the super-resolved results.

The CNN models have achieved excellent results in traditional detection, but they rely too much on the objective function that minimizes the mean square error (MSE). In this way, the PSNR is high, but the reconstructed images are too smooth, and the high-frequency details of the high-resolution images are missing. As the depth of the training network increases, convergence becomes slower and slower, resulting in disappearing gradients, instability, etc. The performance of the generative adversarial network [11] model in image reconstruction surprised researchers. Huang et al. [12] and Goodfellow et al. [13] discussed and compared the importance of GAN models and their variants in generating samples. Creswell et al. [14] provided a survey summary of GAN evaluation methods and training problems. Ledig et al. [15] first applied a generative adversarial network to the field of image super-resolution reconstruction and proposed a generative adversarial network super-resolution reconstruction model (SRGAN), which uses perceptual loss and adversarial loss to jointly recover texture details of images. Bulat et al. [16] designed a GAN based on a High-to-Low network to obtain more natural LR images from HR images to simulate real low-resolution data. Based on SRGAN, Wang et al. [17] proposed an enhanced super-resolution generative adversarial network (ESRGAN) under the condition of improving the network architecture, adversarial loss, and perceptual loss. The network uses dense residual blocks instead of residual blocks in the original generator and uses the relative discriminator to further improve the quality of the reconstructed images. Zhang et al. [18] believed that the process of constructing sample images via down-sampling HR lost the details and accuracy of the original data, so they proposed a super-resolution reconstruction method for real scenes, which has an important impact on subsequent super-resolution reconstruction methods. The SRFlow method proposed by Lugmayr et al. [19] learns image super-resolution through normalized flow and can generate several different SR images from LR images. Rad et al. [20] made adjustments to the composition of perceptual loss and proposed adjusting a target perceptual loss based on object, background, and boundary labels. In particular, Li et al. [21] considered that one-to-one supervision was not the most reasonable way and thus proposed the Beby-GAN with one-to-many supervision. Ma et al. [22] believed that the structure information is very important in the super-resolution problem and proposed implementing an additional gradient branch network. This network generates a high-resolution gradient map as an additional structure prior, making the generated images clearer.

However, most existing networks ignore the feature correlation of adjacent layers and the utilization of some low-frequency information, which leads to the insufficient use of features at different levels. In view of the problems of artifacts and insufficient detail processing in existing algorithms, we propose a differential value dense residual block as the basic unit of the generator to solve the above problems on the basis of improving the generator network structure. At the same time, we use the relative discriminator rather than the standard discriminator, which can further enhance the performance of the generator. Additionally, the average absolute error loss, adversarial loss, and the new joint perceptual loss function are used to train the generator so that the reconstruction performance of the network can reach the best.

The main contributions of this paper are itemized as follows:

1. The use of the traditional convolutional neural networks for image reconstruction neglects the utilization between adjacent layers of the network, which leads to poor image quality. In order to solve this problem, we propose a differential value dense residual network to improve the utilization between adjacent layers of the network;
2. In order to make the training process of the GAN stable and the perceptual quality of reconstructed images better, we introduce a joint perceptual loss function. The loss function uses different layers of the VGG network to extract information with different concerns and fuses high-frequency information and low-frequency information to more effectively guide the network training;
3. To verify the validity and wideness of the proposed method in this paper, we used the proposed model to conduct quantitative and qualitative studies under four datasets and to perform relevant ablation studies.

## 2. Methods

Based on ESRGAN, we propose a differential value dense residual super-resolution reconstruction network model (DVDR-SRGAN), which consists of an improved generator and a discriminator. The generator network is mainly composed of multiple differential value dense residual blocks (DVDB). Each DVDB is composed of differential value structure (DV) and dense residual block (DB). Therefore, the generator can extract the data features from two perspectives. On the one hand, the differential value structure is used to obtain the lost information of each convolution layer; on the other hand, the dense residual block is used to enrich the input information of each deconvolution layer. Then, the information extracted is combined to generate the data that can “cheat” discriminator under the effects of joint perceptual loss, adversarial loss, and mean absolute error loss.

### 2.1. Network Model of DVDR-SRGAN Generator

**Figure 1:**
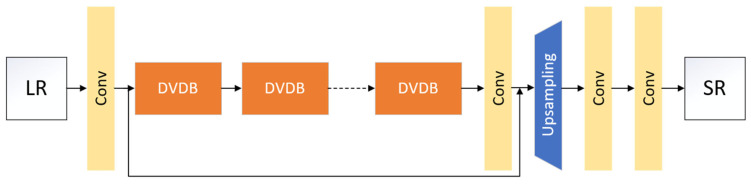
Network model of DVDR-SRGAN generator.

Based on the idea of deconvolution and differential values, the network structure of the generator network model is improved, and the generator network model is shown in Figure 1, which is divided into three parts: shallow feature extraction block, differential value dense residual block set, and image reconstruction.

#### 2.1.1. The Extraction Block of Shallow Feature

The extraction block of shallow feature consists of a convolutional layer, which is represented by Conv. We first input a low-resolution image $I_{LR}$, and then obtain the shallow features of the $I_{LR}$ image by extracting block of shallow features, as shown in the following Formula (1):

(1) $$F_{in}=f_{T}(I_{LR}),$$

where $f_{T}(\cdot )$ represents the operation of the shallow feature extraction block, and $F_{in}$ represents the shallow feature of the $I_{LR}$ image.

#### 2.1.2. A Set of Differential Value Dense Residual Blocks

**Figure 2:**
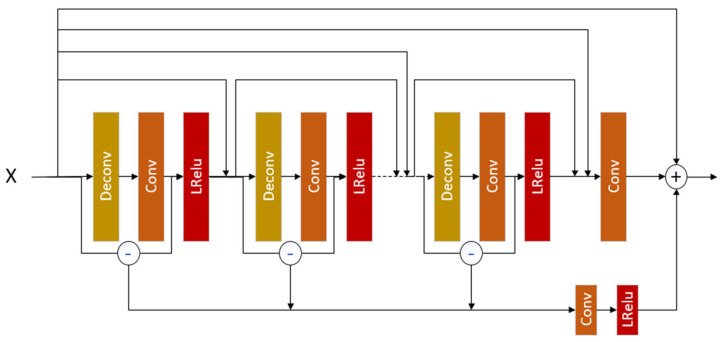
dvdb network structure diagram.

Convolutional neural network extract features from images by means of layer-by-layer sampling. If the area mapped by the pixel on the output feature of the convolution layer on the input is too small, that is, the receptive field is too small, then you can only focus on the local feature. If the receptive field is too large, it contains too much useless information. In order to effectively use the features of each layer and strengthen the feature transmission between each layer, we introduce the differential value dense residual technology into the convolutional network. The dvdb network structure is designed as shown in Figure 2, where Deconv represents deconvolution layer, Conv represents convolution layer, and LRelu represents the Leaky ReLu activation function. First, the dense residual technique is used so that the input of each deconvolution layer is a stitching of the input of the previous deconvolution layer and the output of the corresponding convolution layer. The introduction of this structure allows each layer to make direct use of the gradient information and input information of the loss function, thus alleviating the gradient disappearance phenomenon to a certain extent and helping to train deeper networks. Secondly, in order to avoid only paying attention to local features during feature extraction, a differential value structure is introduced. Feature extraction is carried out according to the differential value, and the extracted features are given corresponding weights, thus enhancing the ability of information extraction and improving the network performance and accuracy. Based on the dvdb network structure, a set of differential value dense residual blocks is designed, which is composed of multiple DVDB modules. Each DVDB module contains three dvdb modules.

In the dvdb network structure, the low-resolution image x is first sent to Deconv, and then Deconv amplifies the features of x to obtain more high-frequency information. Next, the output $F_{decon}^{i}(x)$ of Deconv is input into conv, and the size of the output $F_{con}^{i}(x)$ of conv is guaranteed to be the same as the size of x. In order to prevent information loss and network failure to train as the network increases in depth, we adopt dense residual technology [23]. This technique compensates for lost information by fusing x and the output $F_{con}^{i}(x)$ of conv. As the fused information becomes more reflective of the original high-frequency information, some of the lower-frequency information about the image texture is ignored. In order to retain low-frequency information, we make a difference between x and $F_{con}^{i}(x)$ to obtain the differential value between them. This difference in value can enrich the acquired image information. We sum the differential values of each group of Deconv input and conv output in dvdb and then pass the sum value through a conv and an LRelu successively. The result obtained is weighted with the dense residual result to obtain the final result of dvdb.

(2) $$F_{\mathrm{d-value}}=\sum_{i=1}^{2}F_{in}^{i-1}-F_{con}^{i}(F_{decon}^{i}(F_{in}^{i-1}))$$

(3) $$F_{dvdb}=\alpha F_{\mathrm{d-value}}+\beta F_{db}$$

(4) $$F_{out}=F_{dvdb}(F_{in})+F_{in}$$

where $F_{\mathrm{d-value}}(\cdot )$ represents the sum of differential values in dvdb. $F_{decon}^{i}(\cdot )$ and $F_{con}^{i}(\cdot )$ represent the output values of the *i*th deconvolution layer and the *i*th convolution layer, respectively. $F_{db}(\cdot )$ represents the result of dense residual, and $F_{out}(\cdot )$ represents the output of dvbd.

#### 2.1.3. Image Reconstruction

First, the output of the differential value dense residual block set is upsampled, then the upsampled result is passed through two convolution layers in turn, and, finally, the required size SR image is output. This reconstruction method reduces network complexity.

### 2.2. Relative Discriminator Network

**Figure 3:**
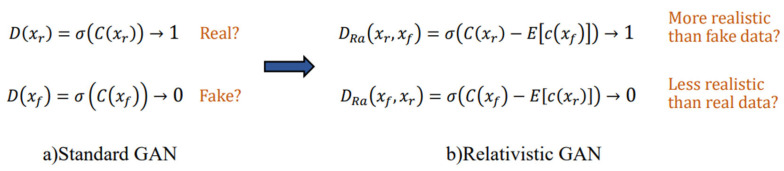
The difference between standard discriminator and relative discriminator.

The traditional discriminator of GAN can only determine the probability that the input image is real and natural under the same network structure, while the relative discriminator is introduced in ESRGAN to try to predict the probability that the real image is more real than the false image, as shown in Figure 3.

**Figure 4:**
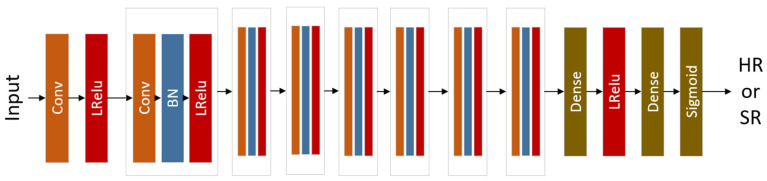
Relative discriminator network model.

In adversarial training, using this discriminator can help the network learn clearer edges and finer textures. Therefore, this paper adopts the relative discriminator RaD of ESRGAN. The specific network model is shown in Figure 4, where Conv represents the convolution layer, LRelu represents the Leaky ReLu activation function, BN represents the batch normalization layer, and Dense represents the full connection layer.

In the relative discriminator network model, Leaky ReLU and BN layers are used simultaneously, and pooling operation is not used. The relative discriminator network consists of 8 convolution layers, all of which are 3 × 3 convolutional kernels in size. The number of convolutional kernels has doubled from 64 to 512. The discriminator network alternately uses convolution layers with step size, which is 1 and 2, respectively. When the number of features doubles, the convolution layer with step size of 2 is used to reduce the image resolution. After the convolution layer obtains advanced features, the probability is finally obtained through two full connection layers and the sigmoid activation function.

The loss function of the relative discriminator and the adversarial loss function of the generator are shown in Formulas (5) and (6):

(5) $$L_{D}^{Ra}=-E_{x_{r}}[\log(D_{Ra}(x_{r},x_{f}))]-E_{xf}[\log(1-D_{Ra}(x_{f},x_{r}))]$$

(6) $$L_{G}^{Ra}=-E_{x_{r}}[\log(1-D_{Ra}(x_{r},x_{f}))]-E_{xf}[\log(D_{Ra}(x_{f},x_{r}))]$$

(7) $$D_{Ra}(x_{r},x_{f})=\sigma (C(x_{r})-E_{xf}[C(x_{f})])$$

where $x_{f}$ represents the input LR image; $x_{r}$ represents the input HR image; $E_{xf}[\cdot ]$ represents the average of the data generated by all generators; σ is a sigmoid function; $L_{D}^{Ra}(\cdot )$ represents the loss of discriminator; $L_{G}^{Ra}(\cdot )$ represents the generator adversarial loss. It can be seen that the adversarial loss of the generator include $x_{r}$ and $x_{f}$. Therefore, in adversarial training, our generator benefits from the gradient between generated data and real data, while in traditional GAN, only the generated data is effective.

### 2.3. Loss Function

The traditional mean square error loss (MSE) is introduced in SRGAN as a part of the loss, while the relative discriminator is introduced in ESRGAN to cancel the mean square error loss, and the pre-activated features are used to construct the perceptual loss to obtain more information. Research [17] shows that the use of pre-activated features will cause the brightness of the reconstructed image to be inconsistent with the real image, so this paper continues to use the pre-activated features. On this basis, a new joint perceptual loss function is proposed, which combines the mean absolute error and adversarial loss to train the network.

#### 2.3.1. Joint Perceived Loss Function

VGG-54 is defined in the deep network, which extracts the feature map of high-frequency features. These feature maps pay more attention to the content. VGG-22 is defined in the shallow network, and most of the extracted features are low-frequency features such as contour and shape [24]. Only using VGG-54 to define the loss will make the texture of the reconstructed image too sharp, which results in distortion of the details and produces noise and artifacts, etc. On the basis of ESRGAN, this paper uses the pre-activation features of VGG-54 and VGG-22 to construct the joint perceptual loss function so that the reconstructed image has the feature of smooth details, offsets the part noise generated, and can also have a good subjective visual effect. The improved loss is shown in Formula (8) as follows:

(8) $$l_{VGG}^{SR}=\alpha *l_{VGG-22}+\beta *l_{VGG-54},$$

where $l_{VGG-22}$ is the feature map before the 2nd convolutional layer before the 2nd pooling in the VGG19 network, which is the ultra-low frequency feature; $l_{VGG-54}$ is the feature map before the 4th convolutional layer before the 5th pooling in the VGG19 network, which is the high-frequency feature; α and β are pre-set parameters. We will conduct the related experiments on the parameter values in Section 3.

#### 2.3.2. Mean Absolute Error Loss

The mean absolute error (MAE) is the average of the absolute error between the predicted value and the observed value. MAE can avoid the error offsetting each other, so it can accurately reflect the actual prediction error. Therefore, this paper combines the average absolute error loss with the joint perceptual loss to increase the prediction effect of the model. The formula of MAE is shown as follows.

(9) $$l_{MAE}=\frac{1}{m}\sum_{i=1}^{m}\left|G(I_{i}^{LR})-I_{i}^{HR}\right|,$$

where $l_{MAE}$ represents the mean absolute error loss function, m is the number of iterations, i is the HR serial number, $I_{i}^{HR}$ is the distribution of the real image, and $G(I_{i}^{LR})$ is the distribution of the high-resolution image generated by the generator.

We fuse the joint perceived loss proposed in Section 2.3.1, the average absolute error, and the adversarial loss given in Section 2.3.2. The improved loss function is shown in the following Formula (10):

(10) $$l_{SR}=l_{VGG}^{SR}+\eta l_{MAE}+\gamma l_{Gen}^{SR},$$

where $l_{Gen}^{SR}$ represents adversarial loss, which can make the images reconstructed by generator network deceive the discriminator network as much as possible and enhance the expressive force of the reconstructed image in terms of visual perceptual. η and γ are coefficients that balance different loss terms. We will conduct relevant experiments on parameter values in Section 3.4.

## 3. Experiments

### 3.1. Datasets and Evaluation Metrics

The training set used in this paper is the DIV2K dataset [25], which contains 800 images. These images are divided into various types, such as city, architecture, landscape, nature, and so on. These images are all from the real world and have high complexity and authenticity. The test datasets include Set5 [26], Set14 [27], BSD100 [28], and Urban100 [29]. Set5 and Set14 are low-complexity single-image super-resolution datasets. BSD100 contains various degraded images, such as images with noise, blur, and lossless compression. Urban100 is an image super-resolution dataset for urban environments, which contains 100 high-resolution images. These four datasets are commonly used for performance testing of super-resolution tasks. When training the network, first, the high-resolution images in the training set are randomly flipped horizontally or vertically, and then the images are clipped to obtain 128 × 128 high-quality image blocks. Finally, we perform the bicubic interpolation on these image blocks to obtain the down-sampling 4 × LR images for training.

In this paper, we use three evaluation metrics, namely peak signal-to-noise ratio (PSNR), structural similarity (SSIM) [30], and learned perceptual image patch similarly (LPIPS) [31] to evaluate the performance of algorithms. As shown in Formula (11), PSNR can evaluate the quality of the image by comparing the gray value difference of the pixels corresponding to the two images. The higher the PSNR value is, the smaller the distortion is. As shown in Formula (12), SSIM evaluates the similarity of the two images from brightness, contrast, and structure, and the closer the SSIM value is to 1, the more similar the structure of the reconstructed image is to the original image, and the better the reconstructed effect. As shown in Formula (13), the LPIPS metric can measure the difference between two images. The lower the value of LPIPS, the more similar the two images are.

(11) $$PSNR(X,Y)=10*\mathrm{lg}\frac{255^{2}*w*h*c}{\sum_{m=1}^{w}\sum_{n=1}^{h}\sum_{z=1}^{c}{\left[X(m,n)-Y(m,n)\right]}^{2}},$$

where X represents the original HR images; Y represents the reconstructed SR images by the generator; c represents the number of channels in the images; w and h represent the width and height of the images; m represents the *m*-th pixel on the width of the image; n represents the *n*-th pixel in the height of the image; z represents the *z*-th channel of three primary color channels.

(12) $$SSIM(X,Y)=\frac{\left(2\mu_{X}\mu_{Y}+C_{1})(2\sigma_{XY}+C_{2}\right)}{\left(\mu_{X}^{2}+\mu_{Y}^{2}+C_{1})(\sigma_{X}^{2}+\sigma_{Y}^{2}+C_{2}\right)},$$

where $\sigma_{X}$ represents the average value of X, and $\mu_{Y}$ represents the average value of Y; $\sigma_{X}^{2}$ represents the variance of X, $\sigma_{Y}^{2}$ represents the variance of Y, and $\sigma_{XY}$ represents the covariance between X and Y; $C_{1}={\left(0.001*L\right)}^{2},C_{2}={\left(0.003*L\right)}^{2}$ are the two variables used to maintain stability, and L represents the dynamic range of image pixels.

(13) $$LPIPS(x,x_{0})=\sum_{l}\frac{1}{H_{l}W_{l}}\sum_{h,w}||w_{l}⊙(y_{hw}^{l}-y_{0hw}^{l})|{|}_{2}^{2}.$$

The real image x and reconstructed image $x_{0}$ are sent to the neural network for feature extraction. The output of each layer is activated and normalized, denoted as $y_{}^{l},y_{0}^{l}\in R^{H_{l}\times }{{}^{W_{l}\times }}^{C_{l}}$. Then, the L2 distance is calculated by multiplying the weight points of the w layer, and the average distance is obtained.

### 3.2. Training Details

The parameters of hardware equipment for experiment conduction are Intel(R) Xeon(R) CPU E5-2680 v4, instance memory: 28G, core: 28, 3080 Ti-12G; Experimental environment: Linux, PyTorch 1.12.0(Python3.8), Cuda11.3.

The training process is divided into two stages. First, a PSNR-oriented model is trained using the average absolute error as the loss function. The initial learning rate is set to 2 × 10^−4^. Next, the learning rate reduces to 0.5 times after each 5 × 10^4^ iteration. Then, the perceptual loss and adversarial loss are introduced into the PSNR-oriented model to obtain the final model. The initial learning rate is set to 1 × 10^−4^. Next, the learning rate reduces to 0.5 times after each 5 × 10^4^ iteration. In the training process, the model uses the Adam optimizer, the batch_ size is set to 16, and the generator network uses 18 DVDB feature extraction blocks. The trained PSNR-oriented model is used as the pre-trained model. One reason is to make the GAN more stable and avoid the remaining local optima in the generator, and the other reason is to ensure that the images received by the discriminator have high resolution, which helps the discriminator to focus more on texture recognition.

In order to obtain the values of α and β in Formula (8), we conducted the experiments on public test sets Set5 and Set14, and the experimental results are shown in Table 1. When the values of α and β are 0.2 and 0.8, respectively, the performance of PSNR, SSIM, and LPIPS reaches the best.

### 3.3. Comparative Experiments

In this paper, our proposed algorithm DVDR-SRGAN is compared with Bicubic, SRGAN [15], ESRGAN [17], Beby-GAN [21], and SPSR [22] on the public sets Set5, Set14, BSD100, and Urban100. In this paper, we evaluate the algorithm performance based on the quantitative results and qualitative results, as follows.

#### 3.3.1. Quantitative Results

Table 2 shows the comparison results of the proposed algorithm with other comparative algorithms on multiple evaluation metrics. The algorithm DVDR-SRGAN has the highest PSNR and SSIM values on the Set5, Set14, and BSD100 datasets, and is also outstanding in LPIPS. Only when facing the Urban100 dataset, the metric results are slightly lower than the algorithm SPSR. The algorithm SPSR based on gradient loss also has better PSNR and SSIM values, but the LPIPS value is not as good as the algorithm DVDR-SRGAN, which shows that the visual quality of the image generated by the algorithm SPSR is much worse than that of the algorithm DVDR-SRGAN. The algorithm Beby-GAN performs poorly in reconstructing large images. The main reason is that the algorithm Beby-GAN uses a method with one-to-many supervision. All the values of evaluation metrics of the algorithm ESRGAN are not as good as algorithm DVDR-SRGAN, which indicates that the algorithm ESRGAN is at the expense of authenticity to optimize the visual quality of the reconstructed images. All the values of evaluation metrics of algorithm SRGAN and algorithm Bicubic are poor, indicating that satisfactory results cannot be obtained when facing tasks of high-quality image super-resolution. In summary, the algorithm DVDR-SRGAN can effectively solve the problem of the general lack of authenticity in adversarial perception methods and ensure the visual quality as much as possible while increasing image details.

#### 3.3.2. Qualitative Results

**Figure 5:**
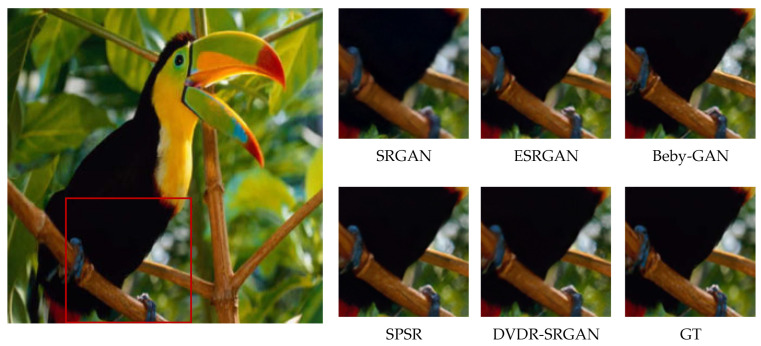
Algorithms 4× reconstruction results. Image “bird” from Set5.

**Figure 6:**
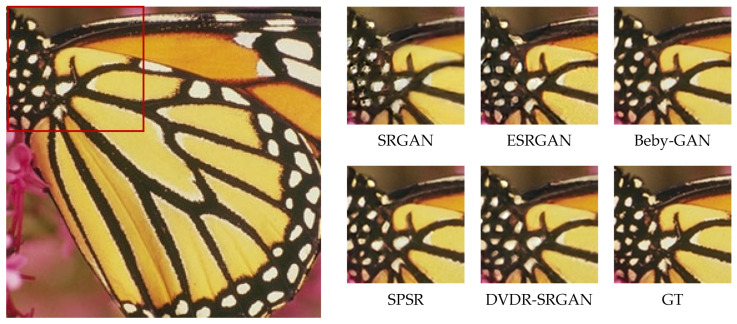
Algorithms 4× reconstruction results. Image “butterfly” from Set5.

**Figure 7:**
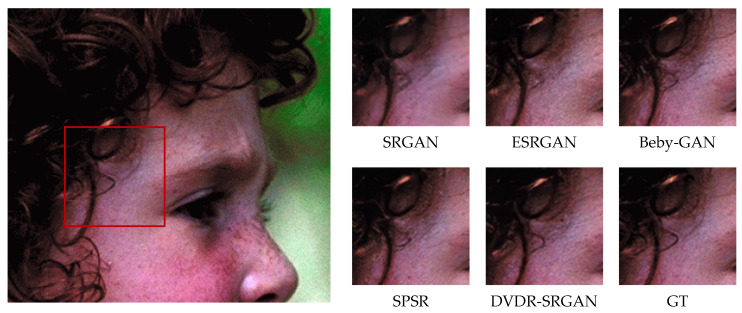
Algorithms 4× reconstruction results. Image “face” from Set14.

**Figure 8:**
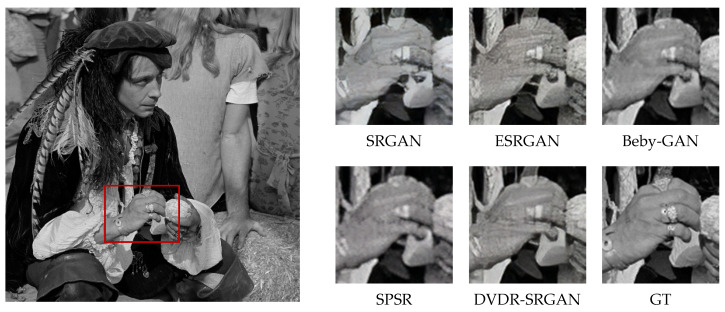
Algorithms 4× reconstruction results. Image “man” from Set14.

**Figure 9:**
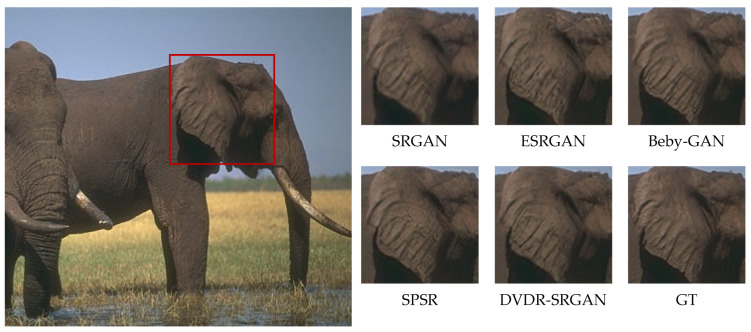
Algorithms 4× reconstruction results. Image “296059” from BSDS100.

**Figure 10:**
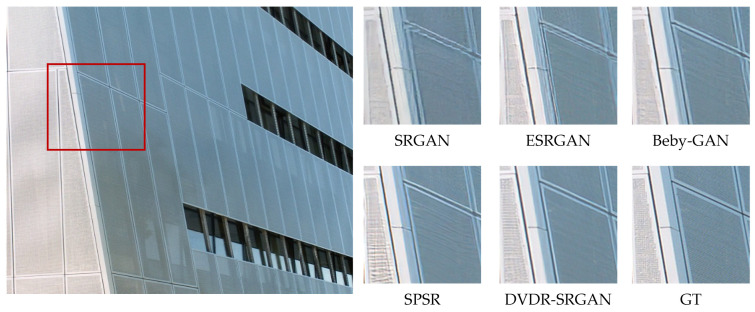
Algorithms 4× reconstruction results. Image “026” from Urban100.

In order to more intuitively feel the visual effect of the algorithm DVDR-SRGAN, we take SRGAN, ESRGAN, Beby-GAN, and SPSR as the comparison algorithms and carry out the comparison experiment based on the four test sets mentioned above. Figure 5, Figure 6, Figure 7, Figure 8, Figure 9 and Figure 10 show some of the results of reconstructed images. In order to better illustrate the reconstruction effect, we take a portion of each image for comparison, as shown in the red box in the image.

From the visual perspective, Figure 5 highlights the image of a parrot’s foot grasping a branch. It can be observed that except for the images of the parrot’s foot generated by DVDR-SRGAN and SPSR, which are more obvious, the images generated by other methods have a certain degree of distortion. However, compared to SPSR, DVDR-SRGAN can still make the branch image in the backward position clear. Figure 6 highlights the butterfly’s back and wing image. The reconstructed image using the algorithm DVDR-SRGAN is closest to GT, while the images generated by other algorithms either have a poor shape or have too many unrealistic artifacts. Figure 7 highlights the hair image near the boy’s temple. For the most obvious strand of hair in the middle, only the image reconstructed by algorithm DVDR-SRGAN is closest to GT, and other algorithms cannot do it. Figure 8 highlights the image of the man’s hand. The reconstructed image using the algorithm DVDR-SRGAN is closest to GT, while the images generated by other algorithms either have a poor shape or have too many unrealistic artifacts. Figure 9 highlights the image of the elephant’s ears. Only the algorithm DVDR-SRGAN can generate dense and clear cross stripes, while other algorithms cannot do it. Figure 10 highlights the pattern of the building. The reconstructed image using the algorithm DVDR-SRGAN is closest to GT, while other algorithms generated unrealistic artifacts when reconstructing the left wall.

In summary, the images generated by the algorithm SRGAN have serious detail loss. In addition to producing too many artifacts in the reconstruction process, the images generated by algorithm ESRGAN also have a loss of details. Due to the use of the method with one-to-many supervision in the algorithm Beby-GAN, it is not possible to perform better image reconstruction when dealing with large-sized images. Algorithm SPSR restores high-resolution gradient maps via a gradient branch to provide additional structure priors for the SR process and introduces the loss function of gradient, which alleviates the problem of image reconstruction distortion. However, a better reconstruction effect cannot be achieved for image areas with small color differences. The algorithm DVDR-SRGAN introduces deconvolution and differential value dense residual methods. This algorithm not only focuses on high-frequency information but also learns and extracts effective features from low-frequency information with weak color to make the reconstructed image more realistic.

### 3.4. Ablation Study

**Figure 11:**
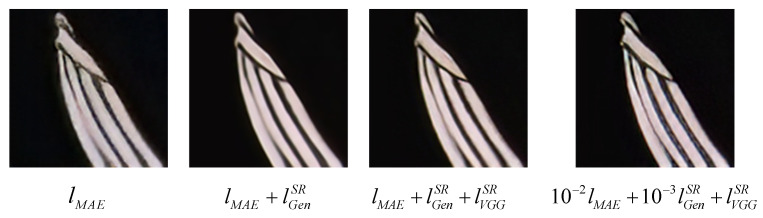
Visual evaluation of different loss functions.

In order to verify the necessity of each part of our proposed network model, we conduct an ablation study on loss function via superposition. Figure 11 shows the relevant visual effects. With the superposition of the loss function, the structure of the reconstructed images is gradually clear, and the artifacts are eliminated, thus improving the authenticity and visual quality. When $\eta =10^{-2},\gamma =10^{-3}$, the reconstructed images have the best effect.

As algorithm DVDR-SRGAN is proposed based on model ESRGAN, we conducted an ablation study on network modules. We designed three algorithms to compare them. The first algorithm (DVDB no JPL) is trained without using joint perceived loss, but the DVDB network module is applied in the network. The second algorithm (DVDB no dvdb) has the same network model as ESRGAN and is trained using joint perceived loss. The third algorithm (DVDB) uses our proposed model. The experimental results are shown in Table 3.

From Table 3, it can be seen that compared with ESRGAN, the network with DVDB has significantly improved the LPIPS performance, which proves the effectiveness of the proposed differential value dense residual in improving the perceptual quality. In addition, the experiment results of the network model with joint perceptual loss show that joint perceptual loss can significantly improve the performance of images in PSNR and SSIM. For algorithm, DVDR-SRGAN, all the values of evaluation metric are better than algorithm ESRGAN on different test sets. Therefore, the effectiveness of our proposed method has been clearly verified.

## 4. Conclusions

Aiming at the super-resolution task with high visual quality, this paper first proposes DVDR-SRGAN, a differential value dense residual network, which can obtain more effective information in the process of feature extraction and improve learning efficiency. Additionally, a relative discriminator has also been introduced to replace traditional discriminator, which can obtain a more realistic probability of real images compared to false images, thereby promoting the reconstructed images via the generator to have more detailed textures. Then, the joint loss function is used to merge the high-frequency information and low-frequency information, which improves the visual effect of the reconstructed images to some extent. Finally, a large number of quantitative and qualitative experiments verify the effectiveness of the proposed method, and the necessity of the proposed method is verified by the ablation study. In future work, considering the complexity of the network, we will focus on optimizing the computational complexity of the network and trying to build a high-performance lightweight network.

## Figures and Tables

**Table 1:** Comparison of values of α and β. The best performance is highlighted in red (1st best).

Parameter		Set5			Set14		
α	β	PSNR	SSIM	LPIPS	PSNR	SSIM	LPIPS
0.1	0.9	28.40	0.796	0.061	24.69	0.602	0.107
0.2	0.8	28.52	0.825	0.052	24.81	0.702	0.094
0.3	0.7	27.93	0.767	0.068	24.56	0.653	0.110
0.4	0.6	27.85	0.759	0.073	24.53	0.621	0.121

**Table 2:** Comparison of PSNR, SSIM, and LPIPS values of 4× reconstruction results of each algorithm. The best performance is highlighted in red (1st best) and blue (2nd best).

Dataset	Metric	Bicubic	SRGAN	ESRGAN	Beby-GAN	SPSR	DVDR-SRGAN
Set5	PSNR	26.66	26.91	27.35	27.82	28.44	28.52
	SSIM	0.790	0.804	0.806	0.801	0.824	0.825
	LPIPS	0.364	0.131	0.108	0.118	0.087	0.064
Set14	PSNR	24.23	23.87	23.61	24.69	24.75	24.81
	SSIM	0.685	0.677	0.650	0.701	0.696	0.702
	LPIPS	0.387	0.142	0.125	0.109	0.106	0.098
BSD100	PSNR	22.65	22.67	23.33	24.13	24.21	24.21
	SSIM	0.601	0.636	0.613	0.635	0.655	0.667
	LPIPS	0.445	0.163	0.143	0.119	0.119	0.118
Urban100	PSNR	21.70	21.77	21.82	22.75	23.24	22.91
	SSIM	0.652	0.677	0.679	0.695	0.737	0.706
	LPIPS	0.435	0.153	0.135	0.109	0.106	0.108

**Table 3:** Comparison of methods under different network modules.

Method	Set14			BSD100			Urban100		
	LPIPS	PSNR	SSIM	LPIPS	PSNR	SSIM	LPIPS	PSNR	SSIM
ESRGAN	0.138	23.61	0.650	0.147	23.33	0.613	0.178	21.82	0.679
DVDB no JPL	0.128	24.25	0.648	0.138	23.59	0.601	0.171	21.99	0.671
DVDB no dvdb	0.131	24.52	0.657	0.142	24.04	0.623	0.176	22.29	0.681
DVDR-SRGAN	0.122	24.81	0.668	0.132	24.21	0.627	0.168	22.91	0.706

## Data Availability

Our training set DIV2K datasets can be obtained from available online: https://data.vision.ee.ethz.ch/cvl/DIV2K/ (accessed on 21 November 2022). Set5, Set14, BSD100, Urban100 can be obtained from: https://arxiv.org/abs/1909.11856/ (accessed on 21 November 2022).
